# Is there any truth in the myth that IVF treatments involve weight gain?

**DOI:** 10.3389/frph.2023.1327110

**Published:** 2024-01-08

**Authors:** Bozhena Saar-Ryss, Michael Shilo, Michael Friger, Leonti Grin, Yulia Michailov, Simion Meltcer, Svetlana Zaks, Jacob Rabinson, Tal Lazer, Shevach Friedler

**Affiliations:** ^1^ IVF Unit, Barzilai University Medical Center, Ashkelon, Israel; ^2^ Faculty of Health Sciences, Ben-Gurion University of the Negev, Beer Sheva, Israel; ^3^ Department of Epidemiology, Biostatistics and Community Health Sciences, Faculty of Health Sciences, Ben-Gurion University of the Negev, Beer-Sheva, Israel

**Keywords:** IVF, antagonist protocol, weight gain, weight change, COH

## Abstract

**Purpose:**

To examine body weight change in women undergoing *in vitro* fertilization and embryo transfer (IVF-ET) using antagonist protocol after up to three treatment cycles.

**Methods:**

A prospective cohort study among IVF patients treated between 2018 and 2019. Each patient underwent weight measurement three times during the treatment cycle: before treatment, at the beginning of the hormonal stimulation, and at the completion of the cycle, on the day of the pregnancy test. Data were also analyzed according to the body mass index (BMI) groups for normal weight, overweight, and obese patients. Finally, weight changes were recorded following altogether 519 treatment cycles, 240, 131, and 148 cycles, for normal weight, overweight, and obese patients, respectively.

**Results:**

The change in the patient's weight was clinically non-significant either during the waiting period or during gonadotropin administration, and overall, during the first, second, or third treatment cycles. The recorded mean total weight change of 0.26 ± 1.85, 0.4 ± 1.81, and 0.17 ± 1.7, after the first, second, or third treatment cycles, represent a change of 0.36%, 0.56%, and 0.23% of their initial weights, respectively. This change of less than 1% of the body weight falls short of the clinically significant weight gain of 5%–7%. Analyzing the data for the various BMI groups, the changes observed in body weight were under 1%, hence with no clinical significance.

**Conclusion:**

The findings of the study reject the myth that hormone therapy involves clinically significant weight gain, and this can lower the concerns of many patients who are candidates for treatment of assisted reproductive technology.

## Introduction

There is a very prevalent “myth,” among women seeking fertility treatments in general and *in vitro* fertilization (IVF) in particular, that hormonal treatment involving controlled ovarian stimulation (COS) causes an increase in body weight. In discussing the side effects of IVF treatment, women often ask whether hormonal treatment will cause obesity. Concrete, evidence-based information is needed to allow accurate consultation as it may help cope with this concern. However, few studies have studied objective physical changes in body weight during IVF treatment to date (1, 2).

IVF medications can potentially lead to significant weight gain during COS (2), although there is limited data available to substantiate this relationship.

It is noteworthy that in the field of reproductive endocrinology it is not common practice to monitor a woman's weight status throughout either stimulation or transfer cycles. Consequently, published side effects of COS seldom include the possibility of weight gain, and COS medications are not typically categorized as weight-promoting medications (3).

Considering that obesity can often lead to unfavorable IVF outcomes (4, 5), there exists a clinical imperative to provide better support to women who may experience weight gain as a result of IVF treatment and to optimize weight management throughout a woman's reproductive journey.

In a previous study, statistically significant weight gain was observed in women undergoing IVF treatment. This weight gain was found to be proportional to the number of oocytes retrieved and was independent of factors such as age, body mass index (BMI), stimulation protocol, or gonadotrophin dose (2, 6, 7). However, it is important to note that the weight gain reported, which amounted to 600 g or approximately 1.3 pounds, was determined to be clinically irrelevant and attributed to temporary edema.

In a more recent study, an average increase of 2.2 kg, equivalent to 4.9 pounds, was documented during ovulation induction in patients with polycystic ovary syndrome (PCOS). This increase in weight was more pronounced in individuals with higher BMI and those who underwent three or more IVF cycles, indicating that these factors may contribute to greater weight gain (8).

However, the consistent and sudden weight experienced by the patient with each IVF cycle strongly suggests that COS may have played a pivotal role. One plausible explanation for this weight gain could be linked to the patient's use of steroid hormone contraceptives, which contain elevated doses of estrogen and progestin (9–11). COS, as part of IVF treatment, leads to substantial increases in estradiol and progesterone levels, often surpassing those observed during pregnancy. The potential for fertility drugs to influence metabolic or hormonal pathways, both in the short term and, possibly, long term, warrants further investigation.

The aim of our study was to investigate potential changes in a patient's body weight resulting from hormonal stimulation used in IVF. We sought to elucidate whether these weight fluctuations could be attributed to variations occurring during the course of hormonal therapy or during the inter-treatment waiting periods. We also explored the possibility that changes in eating habits, influenced by the mental stress experienced during these intervals, could contribute to observed weight alterations.

## Material and methods

### Study design

A prospective cohort study was carried out that tracked body weight among IVF patients treated at the Barzilai University Medical Center's IVF unit, using an antagonist protocol, between 2018 and 2019. The study was approved by the institutional Helsinki committee, approval number 0046-14-BRZ. Before each treatment cycle, patients underwent a consultation at the IVF unit’s clinic, for planning of the treatment cycle. The patients were treated using a routine gonadotropin-releasing hormone (GnRH) antagonist protocol. Controlled ovarian hyperstimulation (COH) protocol was started on the second day of the cycle using variable daily doses between 150 and 450 IU/day, depending on the patient's age and/or ovarian response in previous cycles. The continuing daily dose was adjusted according to patients’ individual ovarian responses followed by the addition of the GnRH antagonist (0.25 mg/day, cetrorelix, Cetrotide, Serono International SR, Geneva, Switzerland; or Orgalutran. NV Organon, Oss, The Netherlands) when serum E2 levels exceeded 400 pg/ml or a follicle of 13–14 mm appeared. The triggering of the final follicular maturation was performed as soon as three or more follicles were >17 mm in diameter. Oocyte retrieval took place 36 ± 2 h after triggering of final follicular maturation. The oocytes could be inseminated by IVF or intracytoplasmic sperm injection (ICSI), using ejaculated sperm by either partner or donor. The patients came for a pregnancy test after 14 days of the embryo transfer (EF).

Data on patient age and infertility-treatment-related variables were collected from the computerized clinical files.

To differentiate the effect of active hormonal therapy and the effect of the stress during the waiting periods between treatments, each patient underwent weight measurement three times during each treatment cycle: first at the consultation before treatment, then at the beginning of the hormonal stimulation, and finally at the completion of the cycle, on the day of the pregnancy test. All patients consented to the study. The weight was measured by the nurses in the IVF unit using the same instrument, with the patients wearing light clothes and no shoes. Patients who returned for the second or third treatment cycle underwent the same weight measurement protocol.

Only patients for whom all three weight measurements were recorded were included in the study. No changes in diet were observed among patients during the treatment.

The patients included in the study were divided into three groups according to their basic BMI: normal weight (BMI < 25), overweight (BMI 25–30), and obese (BMI > 30), (the latter group included a few patients with morbid obesity). The changes in BMI/body weight were analyzed accordingly.

### Statistical analysis

Quantitative variables are presented as mean and standard deviation (SD) as well as median and range from minimal to maximal (Min–Max). For univariate analysis, statistical differences were calculated with the Mann–Whitney *U* test for non-normal distributed variables. The paired samples were compared using the Wilcoxon non-parametric test for independent samples, as well as the paired Student's *t*-test. *p* <0.05 was considered statistically significant. Analyses were performed using SPSS software v26.0 (IBM, Armonk, NY, USA). The power value was calculated using the Power and sample size program v3.0 (Vanderbilt University, Nashville, TN, USA).

We analyzed the data of 346 subjects, of which 130 went through the second cycle of IVF treatment, and 43 of those went through the third cycle of treatment. Repeated measures of patients were taken. A clinically significant weight gain may be considered as a change of at least 5%–7% of the patient's initial body weight (12, 13). The data analysis indicates that the difference in weight of matched pairs is normally distributed with a standard deviation of 16.58. If the true difference in the mean weight of matched pairs is 4.94, we will be able to reject the null hypothesis that this weight difference is zero with probability (power) 1.000. The type I error probability associated with the test of this null hypothesis is 0.05.

## Results

Overall, the study included 346 patients who underwent one therapeutic cycle, 130 who underwent two cycles, and 43 who underwent three cycles. Out of the 346 patients included in our study, 47.7%, 24.3%, and 28% were normal weight, overweight, and obese, respectively. Finally, weight changes were recorded following 519 treatment cycles, 240, 131, and 148 cycles for the normal weight, overweight, and obese patients, respectively.

The study population characteristics after one, two, and three treatment cycles are presented in Table 1.

**Table 1:** Study population characteristics after one, two, and three treatment cycles.

		1st cycle	2nd cycle	3rd cycle
		(*n* = 346)	(*n* = 130)	(*n* = 43)
Age	Mean ± SD	34.86 ± 5.94	36.03 ± 6.09	36.85 ± 6.28
	Median (Min, Max)	35 (21, 47)	36 (22, 46)	37 (22, 46)
Height	Mean ± SD	162.39 ± 6.85	161.99 ± 6.95	163.88 ± 5.67
	Median (Min, Max)	162 (133, 183)	162 (133, 178)	163 (152, 178)
Weight	Mean ± SD	70.58 ± 16.58	71.07 ± 17.04	73.64 ± 19.02
	Median (Min, Max)	67 (37.9, 118)	67 (40, 119.6)	70 (48.2, 118)
BMI	Mean ± SD	26.77 ± 6.13	27.1 ± 6.39	27.44 ± 7.08
	Median (Min, Max)	25.31 (16.23, 44.04)	26.11 (16.95, 46.72)	26.08 (17.39, 44.3)
Total dose of gonadotropins	Mean ± SD	2,230 ± 1278	2319.7 ± 1359	2395.5 ± 1269
		1,830 (300, 7875)	1,931 (270, 7875)	2,025 (870, 6000)

No significant differences were found comparing the parameters of patients (age, height, weight, BMI, and the total dose of gonadotropins administered) who underwent one, two, or three cycles.

To note, during all cycles examined, a significant weight gain or loss of 7% was observed only in 1.98% and 0.54%, of the cycles, accordingly.

The parameters of the patient's weight measurements, and change, during the waiting period, during gonadotropin administration, and overall, after one, two, and three treatment cycles are presented in Table 2.

**Table 2:** Parameters of the patient's weight measurements, and change, during the waiting period, during gonadotropin administration, and overall, after one, two, and three treatment cycles.

		First cycle	Second cycle	Third cycle
		*n* = 346)	(*n* = 130)	(*n* = 130)
First weight	Mean ± SD	70.58 ± 16.58	71.07 ± 17.04	73.64 ± 19.02
	Median (Min, Max)	67 (37.9, 118)	67 (40, 119.6)	70 (48.2, 118)
Second weight	Mean ± SD	70.78 ± 16.51	71.21 ± 17.17	73.77 ± 19.24
	Median (Min, Max)	67.35 (37.9, 118)	67.5 (41.5, 119.6)	68.9 (48, 118)
Third weight	Mean ± SD	70.84 ± 16.56	71.47 ± 17.52	73.81 ± 19.15
	Median (Min, Max)	67.65 (37.8, 118)	68.05 (41, 122)	71 (47, 120)
Weight change during waiting	Mean ± SD	0.2 ± 1.42	0 ± 0.02	0 ± 0.02
	Median (Min, Max)	0 (−5.5, 8)	0 (−0.05, 0.05)	0 (−0.06, 0.04)
Weight change during hormonal treatment	Mean ± SD	0.06 ± 1.32	0.26 ± 1.39	0.04 ± 1.26
	Median (Min, Max)	0 (−4.1, 4.7)	0.2 (−3.2, 4.5)	0 (−3.3, 3.3)
Total weight change	Mean ± SD	0.26 ± 1.85	0.4 ± 1.81	0.17 ± 1.7
	Median (Min, Max)	0.2 (−7.5, 9.8)	0.45 (−4.1, 5.9)	0.1 (−5.3, 5.2)
*p*		0.83	0.85	0.96

To assess whether there are differences in weight change (third cycle weight − first cycle weight delta) in different cycles of the treatment, the Wilcoxon signed-rank test for related samples was used owing to the non-normal distribution. Statistical significance was found in weight changes during the first and second cycles.

Although having statistical significance, these effects are clinically insignificant, all consisting of less than 1% weight mean change (the clinically significant effect is considered above 5% for this research). The change in a patient's weight was clinically non-significant either during the waiting period or during gonadotropin administration, and overall, during the first, second, or third treatment cycles. The recorded mean total weight change of 0.26 ± 1.85, 0.4 ± 1.81, and 0.17 ± 1.7, after the first, second, or third treatment cycles, respectively, represents a change of 0.36%, 0.56%, and 0.23% of their initial weight, as presented in Table 2.

The patients’ characteristics according to the BMI group during the first, second, and third treatment cycles are presented in Tables 3A–C.

**Table 3:** Patients characteristics according to the BMI group during the (**A**) first, (**B**) second, and (**C**) third treatment cycles.

		Overall	Normal weight	Overweight	Obese	*p*-value
		(*n* = 346)	(*n* = 165, 47.7%)	(*n* = 84, 24.3%)	(*n *=* *97, 28%)	
(A) First treatment cycle						
Age	Mean ± SD	34.86 ± 5.94	34.33 ± 5.91	35.11 ± 5.6	35.49 ± 6.27	0.276
	Median (Min, Max)	35 (21, 47)	34 (22, 47)	35 (23, 46)	35.5 (21, 45)	
Height	Mean ± SD	162.39 ± 6.85	162.95 ± 7.11	161.88 ± 6.86	161.88 ± 6.38	0.196
	Median (Min, Max)	162 (133, 183)	163 (133, 183)	161 (147, 178)	162 (150, 176)	
Weight	Mean ± SD	70.58 ± 16.58	57.54 ± 7.3	71.97 ± 7.09	91.55 ± 10.75	>0.001
	Median (Min, Max)	67 (37.9, 118)	58 (37.9, 77.9)	71.5 (58.3, 88.6)	92 (70, 118)	
BMI	Mean ± SD	26.77 ± 6.13	21.64 ± 2.16	27.42 ± 1.49	34.92 ± 3.56	>0.001
	Median (Min, Max)	25.31 (16.23, 44.04)	21.94 (16.23, 24.96)	27.46 (25.04, 29.98)	34.44 (30.04, 44.04)	
GN total dose	Mean ± SD	2,230 ± 1,278	1,770 ± 859.5	2,414 ± 1,510.5	2770.5 ± 1,417.5	0.65
	Median (Min, Max)	1,830 (300, 7,875)	1,500 (300, 4,500)	1,875 (750, 7,875)	2,400 (300, 6,750)	

**Table:** 

		Overall	Normal weight	Overweight	Obese	*p*-value
		(*n* = 130)	(*n *= 54, 41.5%)	(*n *= 40, 27.7%)	(*n *= 36, 28%)	
(B) Second treatment cycle						
Age	Mean ± SD	36.03 ± 6.09	34.6 ± 6.51	37.13 ± 5.29	36.79 ± 6.1	0.158
	Median (Min, Max)	36 (22, 46)	34 (22, 46)	38 (24, 45)	35.5 (25, 45)	
Height	Mean ± SD	161.99 ± 6.95	162.72 ± 7.63	161.68 ± 6.4	161.25 ± 6.54	0.349
	Median (Min, Max)	162 (133, 178)	162.5 (133, 175)	160 (147, 178)	160.5 (150, 174)	
Weight	Mean ± SD	71.07 ± 17.04	56.91 ± 6.88	71.22 ± 7.58	92.14 ± 12.97	>0.001
	Median (Min, Max)	67 (40, 119.6)	57 (40, 70.1)	70.8 (58, 88.7)	91.5 (71.8, 119.6)	
BMI	Mean ± SD	27.1 ± 6.39	21.48 ± 2.08	27.18 ± 1.44	35.43 ± 4.64	>0.001
	Median (Min, Max)	26.11 (16.95, 46.72	22.12 (16.95, 24.35)	26.92 (25.04, 29.97)	34.18 (30.24, 46.72)	
GN total dose	Mean ± SD	2,320 ± 1,359.8	1,785 ± 912	2,476.5 ± 1,641	934.75 ± 1,317	0.002
	Median (Min, Max)	1,931.25 (270, 7,875)	1,743.75 (270, 4,500)	2,025 (720, 7,875)	2,437.5 (885, 5,400)	

**Table:** 

		Overall	Normal weight	Overweight	Obese	*p*-value
		(*n* = 43)	(*n* = 21, 48.8%)	(*n* = 7, 16.3%)	(*n* = 15, 34.9%)	
(C) Third treatment cycle						
Age	Mean ± SD	36.85 ± 6.28	34.9 ± 6.74	38 ± 4.97	39.07 ± 5.61	0.178
	Median (Min, Max)	37 (22, 46)	35.5 (22, 46)	39 (31, 45)	41.5 (28, 45)	
Height	Mean ± SD	163.88 ± 5.67	164.48 ± 5.72	164.43 ± 8.87	162.8 ± 3.78	0.581
	Median (Min, Max)	163 (152, 178)	163 (152, 175)	162 (156, 178)	165 (157, 168)	
Weight	Mean ± SD	73.64 ± 19.02	58.5 ± 7.75	75.66 ± 10.54	93.91 ± 12.87	>0.001
	Median (Min, Max)	70 (48.2, 118)	59.3 (48.2, 72)	75 (65, 89.5)	95 (75.5, 118)	
BMI	Mean ± SD	27.44 ± 7.08	21.61 ± 2.55	27.84 ± 1.2	35.41 ± 4.62	>0.001
	Median (Min, Max)	26.08 (17.39, 44.3)	22.74 (17.39, 24.91)	27.84 (26.08, 29.56)	34.71 (30.09, 44.3)	
GN total dose	Mean ± SD	2,395.5 ± 1,269	1,980.75 ± 838.5	2,430 ± 1,787.25	2,889.75 ± 1,362.75	0.138
	Median (Min, Max)	2,025 (870, 6,000)	1,800 (870, 3,600)	1,740 (1,200, 6,000)	2,475 (1,425, 6,000)	

BMI, body mass index; GN, gonadotropin.

The mean age and height of the patients were with no differences among the different BMI groups. Their weight and BMI differed according to their BMI group. The mean (and median) total dose of gonadotropin administered was highest in the obese group.

Weight changes according to the BMI group during the first, second, and third treatment cycles are presented in Tables 4A–C.

**Table 4:** Weight changes according to the BMI group during the (**A**) first, (**B**) second, and (**C**) third treatment cycles.

		Overall	Normal weight	Overweight	Obese	*p*-value
		(*n* = 346)	(*n* = 165, 47.7%)	(*n* = 84, 24.3%)	(*n* = 97, 28%)	
(A) First treatment cycle						
First weight	Mean ± SD	70.58 ± 16.58	57.54 ± 7.3	71.97 ± 7.09	91.55 ± 10.75	>0.001
	Median (Min, Max)	67 (37.9, 118)	58 (37.9, 77.9)	71.5 (58.3, 88.6)	92 (70, 118)	
Second weight	Mean ± SD	70.78 ± 16.51	57.88 ± 7.49	72.23 ± 7.42	91.47 ± 10.8	>0.001
	Median (Min, Max)	67.35 (37.9, 118)	58 (37.9, 77.6)	71.5 (56, 90)	91.5 (68.4, 118)	
Third weight	Mean ± SD	70.84 ± 16.56	58.01 ± 7.56	72.19 ± 7.59	91.51 ± 11.07	>0.001
	Median (Min, Max)	67.65 (37.8, 118)	57.9 (37.8, 78)	71.5 (56, 92)	91.5 (67.4, 118)	
Weight change during counseling	Mean ± SD	0.2 ± 1.42	0.34 ± 1.24	0.26 ± 1.45	−0.08 ± 1.64	0.448
	Median (Min, Max)	0 (−5.5, 8)	0 (−2.6, 8)	0 (−4.7, 7)	0 (−5.5, 4.7)	
Weight change during treatment	Mean ± SD	0.06 ± 1.32	0.12 ± 1.14	−0.04 ± 1.35	0.03 ± 1.56	0.803
	Median (Min, Max)	0 (−4.1, 4.7)	0 (−3, 4)	0 (−4.1, 3)	0.1 (−4, 4.7)	
Total weight change	Mean ± SD	0.26 ± 1.85	0.47 ± 1.65	0.21 ± 1.84	−0.04 ± 2.12	0.751
	Median (Min, Max)	0.2 (−7.5, 9.8)	0.3 (−3, 9.8)	0.05 (−3.3, 7.2)	0 (−7.5, 5.6)	
% mean change weight		0.36	0.81	0.29	0.04	

**Table:** 

		Overall	Normal weight	Overweight	Obese	*p*-value
		(*n* = 130)	(*n* = 54, 41.5%)	(*n* = 40, 27.7%)	(*n *= 36, 28%)	
(B) Second treatment cycle						
First weight	Mean ± SD	71.07 ± 17.04	56.91 ± 6.88	71.22 ± 7.58	92.14 ± 12.97	<0.001
	Median (Min, Max)	67 (40, 119.6)	57 (40, 70.1)	70.8 (58, 88.7)	91.5 (71.8, 119.6)	
Second weight	Mean ± SD	71.21 ± 17.17	57.03 ± 7.08	71.25 ± 7.8	92.43 ± 13.02	<0.001
	Median (Min, Max)	67.5 (41.5, 119.6)	56.8 (41.5, 72)	69.9 (58.6, 88)	91.7 (71.2, 119.6)	
Third weight	Mean ± SD	71.47 ± 17.52	56.99 ± 7.34	71.64 ± 7.97	92.99 ± 13.37	<0.001
	Median (Min, Max)	68.05 (41, 122)	56.1 (41, 70.4)	69.9 (58.6, 89.8)	92.3 (72.3, 122)	
Weight change during counseling	Mean ± SD	0 ± 0.02	0 ± 0.02	0 ± 0.02	0 ± 0.01	0.495
	Median (Min, Max)	0 (−0.05, 0.05)	0 (−0.05, 0.05)	0 (−0.03, 0.05)	0 (−0.03, 0.04)	
Weight change during treatment	Mean ± SD	0.26 ± 1.39	−0.04 ± 1.22	0.4 ± 1.28	0.57 ± 1.67	0.084
	Median (Min, Max)	0.2 (−3.2, 4.5)	0 (−3, 4)	0.2 (−2.7, 4.5)	0.3 (−3.2, 4.5)	
Total weight change	Mean ± SD	0.4 ± 1.81	0.07 ± 1.4	0.42 ± 1.89	0.86 ± 2.17	0.213
	Median (Min, Max)	0.45 (−4.1, 5.9)	0.25 (−3.4, 2.5)	0.2 (−4.1, 5.9)	0.85 (−3.6, 5.6)	
% mean change weight		0.56	0.12	0.59	0.93	** **

**Table:** 

		Overall	Normal weight	Overweight	Obese	*p*-value
		(*n* = 43)	(*n* = 21, 48.8%)	(*n* = 7, 16.3%)	(*n* = 15, 34.9%)	
(C) Third treatment cycle						
First weight	Mean ± SD	73.64 ± 19.02	58.5 ± 7.75	75.66 ± 10.54	93.91 ± 12.87	<0.001
	Median (Min, Max)	70 (48.2, 118)	59.3 (48.2, 72)	75 (65, 89.5)	95 (75.5, 118)	
Second weight	Mean ± SD	73.77 ± 19.24	58.5 ± 7.73	75.34 ± 11.08	94.42 ± 12.82	<0.001
	Median (Min, Max)	68.9 (48, 118)	58.4 (48, 72.8)	75 (63, 91.3)	96 (76.5, 118)	
Third weight	Mean ± SD	73.81 ± 19.15	58.62 ± 7.9	75.53 ± 9.98	94.27 ± 13.14	<0.001
	Median (Min, Max)	71 (47, 120)	58.8 (47, 73)	74.5 (63, 89)	96 (76.2, 120)	
Weight change during counseling	Mean ± SD	0 ± 0.02	0 ± 0.02	0 ± 0.03	0.01 ± 0.01	0.578
	Median (Min, Max)	0 (−0.06, 0.04)	0 (−0.03, 0.04)	0 (−0.06, 0.03)	0 (−0.01, 0.04)	
Weight change during treatment	Mean ± SD	0.04 ± 1.26	0.12 ± 0.97	0.19 ± 2.02	−0.15 ± 1.27	0.614
	Median (Min, Max)	0 (−3.3, 3.3)	0.4 (−1.8, 1.7)	0.2 (−3.3, 3.3)	−0.3 (−2, 2)	
Total weight change	Mean ± SD	0.17 ± 1.7	0.12 ± 1.15	−0.13 ± 3.26	0.37 ± 1.46	0.9
	Median (Min, Max)	0.1 (−5.3, 5.2)	0 (−3, 2.8)	0.8 (−5.3, 5.2)	0.1 (−2, 2.2)	
% mean change weight		0.23	0	−0.17	0.39	

Further tests were used on the last two cycles, assessing weight change in different BMI groups, using the Wilcoxon signed-rank test for the related samples owing to the non-normal distribution and small sample size. The statistically significant difference was found for the normal weight and the obese groups during the first and second cycles of treatment accordingly. Although having statistical significance, these effects are clinically insignificant, all consisting of less than 1% weight mean change (a clinically significant effect is considered above 5% for this research).

When the patients were analyzed after the first treatment cycle, according to their BMI group, in the normal BMI group a minor gain of 0.47 ± 1.65 g (*p *= 0.010) and in the obese group a loss of 0.04 ± 2.12 was recorded, whereas in the overweight BMI group no statistically significant changes in body weights were recorded. This minor gain/loss of 0.81%, 0.29%, and 0.04% represents a change of less than 1% of the initial body weight, which is clinically non-significant.

After the second treatment cycle, in the obese BMI group a minor gain of 0.86 ± 2.17 g (*p *= 0.025) was observed whereas in the other BMI groups the changes were insignificant. Again, changes in body weight of 0.12%, 0.59%, and 0.93% represent a change of less than 1% of the initial body weight, which is clinically non-significant. After the third treatment cycle, none of the changes in body weight were significant.

Comparing the change in body weight during the waiting period and during hormonal treatment administration, no statistically significant difference was found, in all groups examined.

Further correlation tests were performed to study the relations between variables and to try building a regression model. No mentionable correlation was found.

## Discussion

In discussing the side effects of IVF treatment, women often ask whether hormonal treatment will cause obesity. The feeling of fullness in the abdomen that is sometimes reported as a side effect of ovarian stimulation can be perceived as evidence of a change in body weight and is confounding, and women who develop ovarian overstimulation do accumulate fluid in the abdominal cavity and gain weight. Still, for many patients, the threat of weight gain poses an obstacle in their decision to start hormonal treatment for ovarian stimulation, especially among candidates for IVF-ET. As the amount of published reliable data is scarce in the literature (1, 2, 14), we decided to conduct a study examining the correlation between exposure to COH for IVF and changes in body weight.

Patients’ weights may be linked to their baseline health (i.e., hypothyroidism and diabetes), the medications administered during treatment, hormonal changes secondary to their ovarian response as well as the changes in their eating habits because of the stress they feel secondary to their infertility and the IVF treatment they must endure. On reviewing the scientific literature, we found a publication, published in 2011, that tracked the weight of 66 IVF patients treated with a long protocol with a GnRH antagonist. They found that their mean weight increased toward the end of the controlled ovarian stimulation but decreased back to their baseline weight at the end of the therapeutic cycle (1). Another publication in 2021 (2) included a retrospective study examining weight change in 734 women undergoing IVF, between the start of the COH treatment and the day of human chorionic gonadotropin (hCG) triggering. Both GnRH agonist and antagonist protocols were used. Although they observed a significant weight gain (a mean of 387.7 ± 720.4 g; *p* < 0.001), regardless of the COH protocol and correlating with the number of oocytes retrieved, they stated that “the weight gain is possibly a result of edema and is clinically irrelevant despite the statistical significance.” Also, the reported weight gain was not correlated to the COH protocol used, and as the difference was measured on the day of hCG administration, no data were given on whether the patients regained their original weight or not at the end of the treatment cycle. In the study by Tso et al. (2), neither the anxiety score (Pearson: *r* = −0.031; *p *= 0.561) nor the binge eating score (Pearson: *r* = 0.069; *p *= 0.199) was correlated with weight gain, based on questionnaires reported by the patients.

In a recent case report (14), a weight gain of 5.89–6.80 kg was reported in a 33-year-old patient with childhood-onset Class II obesity and hypothyroidism between her baseline weight and weight after oocyte pick up (representing about 6% of her initiative weight). This phenomenon occurred in each of the three cycles of the IVF-ET she underwent, during a period of 4 years, although she returned to her basic weight each time.

In our prospective study, only patients treated by a similar GnRH antagonist protocol were included. Weight was recorded during up to three consecutive cycles. We measured possible weight change during the waiting period, when no gonadotropins are administered and during the period of ovarian stimulation and embryo transfer, when patients underwent exposure to the medical treatment involved in the whole IVF-ET procedure. As high BMI was suggested to be correlated with weight gain (8), we analyzed our data also by BMI groups.

Our data show no clinically significant impact, either during the waiting period or during the administration of hormonal treatment, on the patient's body weight, after one, two, or three consecutive treatment cycles, irrespective of the gonadotropin dose administered, and irrespective of the patient's baseline BMI group, as the changes in the body weight were all under 1%.

Thus, during our consultations, we aim to alleviate concerns of individuals considering IVF treatment by assuring them that the hormonal treatments required for IVF are unlikely to exert a significant impact on their body weight. It is important to note that our study has some limitations, particularly the relatively small size of the group that underwent two or three treatment cycles. To enhance the robustness of our findings, we recommend further investigations involving a broader spectrum of patients, including those with PCOS and hypothyroidism.

## Conclusions

This is the first prospective study to examine changes in the body weight of women undergoing IVF-ET treatment with an antagonist protocol, both during the days between medical consultation and the onset of hormonal treatment and during actual hormonal treatment, analyzed also by their baseline BMI. The findings of the study reject the myth that hormone therapy involves clinically significant weight gain. This can lower the concerns of many patients who are candidates for treatment of ART.

## Data Availability

The raw data supporting the conclusions of this article will be made available by the authors, without undue reservation.
